# Genetic Determinants of Antibiotic Resistance in *Francisella*

**DOI:** 10.3389/fmicb.2021.644855

**Published:** 2021-05-12

**Authors:** Stephen J. Kassinger, Monique L. van Hoek

**Affiliations:** School of Systems Biology, George Mason University, Manassas, VA, United States

**Keywords:** antibiotic resistance, *Francisella*, tularemia, biofilm, multidrug resistance, antibiotic drug resistance

## Abstract

Tularemia, caused by *Francisella tularensis*, is endemic to the northern hemisphere. This zoonotic organism has historically been developed into a biological weapon. For this Tier 1, Category A select agent, it is important to expand our understanding of its mechanisms of antibiotic resistance (AMR). *Francisella* is unlike many Gram-negative organisms in that it does not have significant plasmid mobility, and does not express AMR mechanisms on plasmids; thus plasmid-mediated resistance does not occur naturally. It is possible to artificially introduce plasmids with AMR markers for cloning and gene expression purposes. In this review, we survey both the experimental research on AMR in *Francisella* and bioinformatic databases which contain genomic and proteomic data. We explore both the genetic determinants of intrinsic AMR and naturally acquired or engineered antimicrobial resistance as well as phenotypic resistance in *Francisella*. Herein we survey resistance to beta-lactams, monobactams, carbapenems, aminoglycosides, tetracycline, polymyxins, macrolides, rifampin, fosmidomycin, and fluoroquinolones. We also highlight research about the phenotypic AMR difference between planktonic and biofilm *Francisella.* We discuss newly developed methods of testing antibiotics against *Francisella* which involve the intracellular nature of *Francisella* infection and may better reflect the eventual clinical outcomes for new antibiotic compounds. Understanding the genetically encoded determinants of AMR in *Francisella* is key to optimizing the treatment of patients and potentially developing new antimicrobials for this dangerous intracellular pathogen.

## Introduction

Antibiotic resistance (AMR) is a significant and emerging threat to modern medicine. AMR, whether naturally acquired or engineered, is also of significant concern in the area of biodefense. In addition, bacteria that have a significant intracellular phase to their lifecycle or form biofilms pose inherent challenges to extracellular antibiotic treatment (phenotypic resistance). The possible emergence of naturally acquired or engineered AMR will only reduce the few therapeutic options available. In light of the emerging AMR crisis and the rapid emergence of antibiotic resistant-bacteria in hospital and community settings, as well as in combat casualty care, it is critical to understand the genetic determinants of AMR.

*Francisella tularensis* is the causative agent of the zoonotic disease tularemia. It is a facultative intracellular bacterium, infecting and replicating within macrophages and other phagocytic cells as well as cells of the reticuloendothelial system in the host. It has been classified as a Tier 1 Category A select agent and a biothreat agent due to its low inhaled infectious dose (>10 organisms for humans) and its historical development as a biological weapon (Dennis et al., 2001). While the disease is relatively uncommon in the current times, outbreaks have become more frequent in recent years in the central United States and across northern Europe (Eliasson et al., 2002; Reintjes et al., 2002; Akalın et al., 2009; Brett et al., 2014; Dupont et al., 2015; Zargar et al., 2015; Coates et al., 2018). *Francisella* is intrinsically resistant to many classes of antibiotics due to the nature of its LPS and the many enzymes it produces (Biswas et al., 2008; Caspar and Maurin, 2017). The facultative intracellular phase of its lifecycle provides additional challenges to systemic antibiotic therapy which can also lead to treatment failures. While no clinical samples have shown enhanced AMR over wild type strains (Urich and Petersen, 2008), this may be a result of the difficulty of isolating *Francisella* from clinical samples (Sutera et al., 2014b) as both the strain and the context of isolation seem to influence the cultivability of *Francisella* (Humrighouse et al., 2011). Nonetheless, several *Francisella* mutants have been isolated against a variety of clinically relevant antibiotics *in vitro*. The genetic determinants of resistance to these drugs are of interest (Munita and Arias, 2016). In recent years, many more strains and species of *Francisella* have been sequenced, enabling this review of the genetic determinants of AMR in *Francisella* and to update earlier summaries (Biswas et al., 2008).

Of critical interest are those drugs that are commonly used to treat patients with tularemia, as enhanced resistance to these would force the use of other less optimal antibiotics. Historically, aminoglycosides such as streptomycin and gentamicin are favored as treatments, followed by tetracyclines such as tetracycline and doxycycline, and more recently fluoroquinolones, such as ciprofloxacin (Dennis et al., 2001).

## Antibiotics Effective Against *Francisella*

Antibiotics work by one of the five general mechanisms: they can (i) inhibit cell wall synthesis, (ii) depolarize the plasma membrane, (iii) inhibit protein synthesis, (iv) inhibit nucleic acid synthesis, or (v) inhibit metabolic pathways. Thus, AMR is usually the result of a genetic change that happens in the bacteria resulting in the ability of the bacteria to survive in the presence of the antibiotic (Reygaert, 2018). Genotypic resistance is therefore inheritable by definition, whereas phenotypic resistance is a change in gene expression that is not heritable. Epigenetics can also be involved in AMR in bacteria (Ghosh et al., 2020, #1868), although this has not been well studied in *Francisella*. We will discuss both genotypic and phenotypic resistance for *Francisella*.

**Research Question:** What is the role of the epigenetics in antibiotic resistance in Francisella?

The antibiotics that are considered most effective against *Francisella* include streptomycin (intramuscular or intravenous), gentamicin (intravenous), doxycycline, tetracycline, and ciprofloxacin (Table 1) (Dennis et al., 2001). Recently, levofloxacin has also shown good results in animal studies (Klimpel et al., 2008; Nelson et al., 2010). Streptomycin and gentamicin are aminoglycoside antibiotics, which are protein synthesis inhibitors, and these two compounds are usually bactericidal. Doxycycline is a “tetracycline class” of antibiotic, and along with tetracycline, works by inhibiting protein synthesis. These antibiotics are usually bacteriostatic. The fluoroquinolone class, ciprofloxacin and levofloxacin, act on DNA Gyrase, thus inhibiting nucleic acid synthesis, and are both bactericidal (Caspar and Maurin, 2017).

**TABLE 1:** Intrinsic antibiotic resistance in *Francisella*.

Antibiotic class	Drug name	*Francisella* response	References
Beta lactam	Penicillin	Resistant	Caspar and Maurin, 2017
Dihydrofolatereductase inhibitors	Sulfamethoxazole trimethoprim (cotrimoxaole)	Resistant	Biswas et al., 2008
Polymyxins	Polymyxin B	Resistant	Caspar and Maurin, 2017
	Colistin (polymyxin E)	Resistant	Caspar and Maurin, 2017
Macrolides	Erythromycin	Sensitive/resistant (strain dependent)	Biswas et al., 2008
Macrolide	Azithromycin	Type B—Resistant, Type A—Sensitive	Ahmad et al., 2010
Carbapenems	Various	Sensitive/resistant (strain dependent)	Tomaso et al., 2005; Hotta et al., 2013; Caspar and Maurin, 2017
Monobactam	Aztreonam	Sensitive/resistant (strain dependent)	Scheel et al., 1993; Garcia del Blanco et al., 2004; Tomaso et al., 2005; Antunes et al., 2012; Dean and van Hoek, 2015; Caspar and Maurin, 2017; Hotta et al., 2020
Cephalosporin	Cefotaxime, moxalactam (latamoxef), ceftazidime	Type A—sensitive, Type B—resistant	Baker et al., 1985; Tomaso et al., 2005
Aminoglycosides	Streptomycin, gentamicin, amikacin, tobramycin	Sensitive	Caspar and Maurin, 2017
	Kanamycin	Sensitive	Zogaj and Klose, 2010
	Hygromycin	Sensitive	Zogaj and Klose, 2010
Aminoglycoside—aminocyclitol	Spectinomycin	Sensitive	Buchan et al., 2008; Zogaj and Klose, 2010; Scarff et al., 2019
Ansamycins (antimycobacterial)	Rifampicin	Sensitive	Caspar and Maurin, 2017
DXR (1-deoxy-D-xylulose 5-phosphate reductoisomerase) inhibitors	Fosmidomycin, FR900098	Sensitive	McKenney et al., 2012
**Clinically used antibiotics**			
Aminoglycosides	Streptomycin, gentamicin	Sensitive	Caspar and Maurin, 2017
Tetracyclines	Tetracycline, doxycycline	Sensitive	Caspar and Maurin, 2017
Fluoroquinolones	Ciprofloxacin, levofloxacin	Sensitive	Caspar and Maurin, 2017

*This table is organized in approximate order of resistance from resistant to mixed to sensitive.*

New antimicrobials are being developed against *Francisella* by several researchers. One class being developed and tested is antimicrobial peptides, which depolarize the plasma membrane of *Francisella*, and may have additional pathogen-directed and host-directed effects as well (Han et al., 2008; Amer et al., 2010; Flick-Smith et al., 2013; Kaushal et al., 2016). Novel antibiotic compounds such as *N*-benzyl aminomethyl spectinomycins (Scarff et al., 2019) and arsinothricin (Nadar et al., 2019) have recently been developed or identified, potentially providing additional therapeutic options for tularemia.

## Mechanisms of Antibiotic Resistance

There are common mechanisms of AMR that are generally found in prokaryotes. These mechanisms include modification of the drug target, limiting the uptake of the drug, inactivation of the drug, and active efflux of the drug.

The bacterium, in response to treatment, can alter the protein sequence of the antibiotic target (Spratt, 1994). This is observed with rifampicin, an antibiotic that has a single known protein target, RpoB which is subject to mutation as a mechanism of resistance (Taniguchi et al., 1996) (see section “Rifampin Resistance”). In another mechanism, some bacteria indirectly interfere with the binding of drug to target protein by way of a blocker protein. This phenomenon is referred to as “target blocking” and while it has been known as a mechanism of resistance for tetracycline for many years (Manavathu et al., 1990), its greater distribution among drug resistances is now becoming more apparent (Wilson et al., 2020). Cells can also overproduce the antibiotic target, effectively overcoming the drug’s inhibition. This is seen with the overproduction of the target protein dihydrofolate reductase in trimethoprim resistance (Huovinen, 2001).

Limitation of uptake of the drug is seen as a mechanism of resistance if the drug-entry into the bacterium is transporter-dependent. For example, we demonstrated that *Francisella novicida* can spontaneously become resistant to fosmidomycin via a mutation in the GlpT (glycerol-3-phosphate) transporter (Mackie et al., 2012), and further that use of a lipophilic prodrug of fosmidomycin can bypass this resistance (McKenney et al., 2012).

Other drug resistance mechanisms focus on the drugs themselves as opposed to the drug targets. A bacterium can destroy the drug as exemplified by penicillins being destroyed by β-lactamses (Majiduddin et al., 2002). A bacterium can also alter the drug such as with chloramphenicol-acetyl-transferase which reduces its activity (Shaw, 1975). More rarely, a bacterium can produce a binding partner for the drug which stochastically limits the influence of the drug such as the case with zeocin resistance and the *ble* gene (Drocourt et al., 1990).

Active efflux of the antibiotic via multidrug efflux pumps is another common mechanism of acquired resistance. This mechanism is commonly seen in Gram-negative pathogens including *Acinetobacter*, *Burkholderia*, and *Pseudomonas* (Kumar et al., 2008; Lee et al., 2017; Zahedi Bialvaei et al., 2021).

## Phenotypic Resistance

Bacteria can exhibit phenotypic resistance to antibiotics based on the expression of a physical characteristic (such as biofilm) or through gaining access to a protected location (such as intracellular replication). *Francisella* employs both of these strategies to demonstrate phenotypic resistance.

A critical aspect in designing antibiotics to eradicate intracellular bacteria is to appreciate the protective effects of their intracellular localization. *Francisella* spp. can be taken up by macrophages, dendritic cells, and other phagocytic cells (Cowley et al., 1997; Golovliov et al., 1997; Bosio and Dow, 2005), as well as hepatocytes, lung epithelial cells, and even red blood cells (Conlan and North, 1992; Gentry et al., 2007; Hall et al., 2007; Schmitt et al., 2017) as a facultative intracellular pathogen. The bacteria are able to use their Type VI secretion system (encoded on the *Francisella* Pathogenicity Island) to escape the phagosome and replicate in the cytosol of the eukaryotic host cell. In this location, the bacteria are phenotypically resistant to many antibiotics that cannot efficiently penetrate the eukaryotic membrane. This is the basis of the “gentamicin protection assay,” for example, in which gentamicin is applied to the infected, cultured host cells, and the intracellular bacteria are unaffected by the antibiotic and can replicate (Isberg and Falkow, 1985). Any extracellular, un-phagocytized bacteria will be killed by the extracellular gentamicin antibiotic, enabling the measurement of intracellular replication (Isberg and Falkow, 1985). This intracellular residence is also thought to contribute to the fairly common relapses observed with bacteriostatic antibiotics such as tetracycline. Interestingly, this apparent “problem” of intracellular localization can also be put to use as a novel screening model to identify antibiotics that are able to affect the intracellular form of *Francisella*. Several of these approaches are summarized at the end of this review.

*Francisella* has been shown to form biofilms (Dean et al., 2009; Durham-Colleran et al., 2010; Margolis et al., 2010; Chung et al., 2014; Champion et al., 2019). *Francisella* embedded in these biofilms were shown to have increase AMR compared to planktonic cells (Biot et al., 2020; Siebert et al., 2020), similar to what has been observed with other bacteria (Singh et al., 2017). This is thought to be mainly due to poor diffusion of the antibiotics through the extracellular polysaccharide matrix that makes up the biofilm. It is not clear what role biofilms play in the *Francisella* lifecycle and/or human disease (van Hoek, 2013). The two-component system that regulates biofilm production (qseBC) is important for virulence, but biofilm is thought to contribute more to survival in *Francisella*’s environmental niches (van Hoek, 2013). Additional mechanisms of bacterial resistance can contribute to the phenotypic resistance provided by the biofilm, including slow growth rate, induction of efflux pump expression, and induction of persister cells (Soto, 2013).

A third example of phenotypic AMR in *Francisella* is demonstrated by the temperature-dependent inhibition of gentamicin uptake at low temperatures (Loughman et al., 2016). This is discussed further in Section “Aminoglycoside Resistance”.

## Horizontal Gene Transfer in *Francisella*

Horizontal gene transfer commonly leads to the acquisition of antibiotic-resistance genes and gene-clusters by Gram-negative bacteria, such as *Acinetobacter* for example (Forsberg et al., 2012; Wang and Sun, 2015). There are three main mechanisms by which DNA transfer occurs in bacteria: bacterial conjugation, natural transformation, and transduction.

• *Conjugation*: Conjugation is the transfer of circular plasmid DNA from one bacterial cell to another through cell–cell contact. This method has been used in *Francisella* to enable the creation of mutants and to mobilize plasmids (Golovliov et al., 2003; Challacombe et al., 2017; Brodmann et al., 2018).
• *Transformation:* Transformation is the introduction of “free” DNA from the environment into a bacterium. In *Francisella*, transformation has been achieved by electroporation and chemical transformation methods, depending on the species (see below).
• *Transduction:* Transduction is the transfer of DNA to a bacterium via a bacteriophage. There have been only a few reports of bacteriophage which can act against *Francisella* so far (Koliaditskaia et al., 1959; Tlapak et al., 2018) and they appear so far to be unstable or difficult to isolate. This area of research is ongoing, but one very interesting finding was the identification of CRISPR/CAS9 system in *F. novicida* (Schunder et al., 2013), suggesting that there have been bacteriophage interactions with *Francisella* in the past, enough to acquire a viral immunity system such as CRISPR/Cas.

In addition to phage and transposons moving DNA, sometimes cells rearrange their DNA though minor failures of normal DNA replication maintenance machinery. The closely related members of the *Francisella* genus all exhibit significant rearrangement of their genomes with respect to each other, suggesting that this has occurred (Petrosino et al., 2006).

## Plasmids in *Francisella*

*Francisella* is unlike many Gram-negative organisms in that it does not appear to carry AMR or virulence genes on plasmids. *Francisella* does not easily acquire plasmids as very few plasmids are identified in isolates (Challacombe et al., 2017). Thus plasmid-mediated acquired AMR does not appear to occur naturally (Challacombe et al., 2017). The few plasmids that have been identified within *Francisella* species do not encode very many proteins, and do not appear to confer any AMR (Pomerantsev et al., 2001a, b; Frank and Zahrt, 2007; Challacombe et al., 2017). *Francisella* cannot express exogenous *Escherichia coli* plasmids (McWhinnie and Nano, 2014) and require the use of *Francisella* promoters and specific codon optimization for high-level plasmid-mediated protein expression (Sjostedt et al., 1990; Golovliov et al., 1997; Brodmann et al., 2018). Thus, it is possible to introduce antibiotic resistance on plasmids experimentally to *Francisella* species for selection during cloning using plasmids typically engineered from pFNL10 or another naturally occurring *Francisella* plasmids as the backbone (Ludu et al., 2008).

Methods of introduction of plasmids used in the laboratory for research purposes include electroporation (Baron et al., 1995) and cryotransformation (Pavlov et al., 1996; Lai et al., 2010) for *F. tularensis*, chemical competence for *F. novicida* (Anthony et al., 1991), and triparental conjugation for *F. tularensis holarctica* LVS (Golovliov et al., 2003; Horzempa et al., 2008) and *F. novicida* (Brodmann et al., 2018).

The antibiotics used for cloning must be those that are not clinically useful for the treatment of tularemia, such as kanamycin (Frank and Zahrt, 2007). The reasons for this are twofold: first, in the unlikely event a researcher contracts *Francisella* in laboratory, that infection can be treated via standard and established methods; and second, due to the historic development of *Francisella* as a biological weapon, creation of a strain resistant to clinically-useful antibiotics could be a violation of the ban on the development of biological weapons. Recently, a new plasmid was constructed to enable tetracycline-inducible protein-expression system for *Francisella* by modifying the promoter to be a strong *Francisella*-specific promoter among other changes (Sheshko et al., 2021) despite tetracycline still being a clinically useful antibiotic for tularemia.

## Intrinsic Antibiotic Resistance in *Francisella*

*Francisella* is intrinsically resistant to many antibiotics (Table 1). This pattern of resistance is predominantly dependent on the genetic determinants of the strains and species, dependent on the expression of chromosomal genes. There are a few instances of phenotypic resistance, discussed above. A study using a comprehensive transposon-insertion library and a phenotype screen in *F. novicida* confirmed the presence of intrinsic antibioitic resistance genes, and identified some genes that were not previously known to be involved in AMR (Enstrom et al., 2012). For example, this study showed very similar antibiotic sensitivities of *acrA/B* and *tolC* mutants, suggesting that these proteins might work together in the resistance to multiple antibiotics.

While *Francisella* does not demonstrate acquired resistance due to mobile elements and horizontal transfer, such as occurs in many human pathogens, it is useful to recognize that this important biothreat pathogen is susceptible to only three major classes of antibiotics and exhibits resistance to many antibiotic classes.

### Beta-Lactam Resistance

Penicillin is known to prevent the growth of many microorganisms (Fleming, 1929) and had been successfully used to treat human staphylococcal infections (Abraham et al., 1941). These results led to the solving of the structure of penicillin and closely related compounds (Chain, 1979) revealing the important structural similarity in the beta-lactam ring. A beta-lactam ring is a four-membered ring consisting of three carbons and a nitrogen with one of the carbons immediately adjacent to the nitrogen being double bonded to an oxygen (Bodey, 1990). Chemists then developed several synthetic derivatives based around the beta-lactam ring (Baldwin et al., 1973; Edwards et al., 1975). During this process, the target for beta-lactams was discovered to be bacterial cell wall synthesis (Strominger et al., 1959). With greater use of beta-lactams came a broader understanding of mechanisms of resistance (Knowles, 1985). Beta-lactam resistance generally is due to one of three different mechanisms: altering outer membrane permeability (LPS), altering the drugs target [penicillin-binding proteins (PBP)], or degrading the drug enzymatically (beta-lactamase) (Bodey, 1990).

Beta lactam antibiotics act against rapidly dividing bacteria by binding to penicillin-binding proteins, and thus inhibiting the bacterial cell wall synthesis. An examination of *Francisella* genomes using the KEGG database reveals four to five potential penicillin-binding protein encoding genes (Table 2), including PBP-3, PBP-4, and PBP5/6 members. Of this group, only one gene, *DacD* (FTN_0907), has been investigated in regard of beta lactam resistance and was shown not to be involved in beta lactam resistance (Spidlova et al., 2018). Interestingly, we have identified a penicillin-binding protein activator (LpoB) in all the *Francisella* genomes examined. This protein is an activator of PBP-1b’s transpeptidase and transglycosylase activities in other organisms, and thus is a regulator of peptidoglycan synthesis. Its role in *Francisella* is unclear given the lack of PBP-1.

**TABLE 2:** The annotated PBP gene in *Francisella* species and subspecies using KEGG database.

	*Francisella tularensis* subsp. *tularensis* Schu S4	*Francisella tularensis* subsp. *holarctica* LVS	*Francisella tularensis* subsp. *novicida* U112
	Locus (KEGG orthology) name [E.C. number], other information	Locus (KEGG orthology) name [E.C. number], other information	Locus (KEGG orthology) name [E.C. number], other information
**Penicillin-binding protein 3 (PBP-3)**	FTT_0697 (K03587) **FtsI** Peptidoglycan D,D-transpeptidase, cell division protein FtsI (penicillin-binding protein 3) [EC:3.4.16.4] **ftsI;** peptidoglycan synthetase	FTL_1539 (K03587) cell division protein FtsI (penicillin-binding protein 3) [EC:3.4.16.4] penicillin-binding protein (peptidoglycan synthetase)	FTN_0607 (K03587) cell division protein FtsI (penicillin-binding protein 3) [EC:3.4.16.4] **ftsI;** cell division protein, peptidoglycan synthetase (PBP)
**PBP-4**	FTT_1039 (K07259) serine-type D-Ala-D-Ala carboxypeptidase/endopeptidase (penicillin-binding protein 4) [EC:3.4.16.4 3.4.21.-] | **dacB1**; D-alanyl-D-alanine carboxypeptidase Uniprot Q5NG20	FTL_1046 (K07259) Serine-type D-Ala-D-Ala carboxypeptidase/endopeptidase (penicillin-binding protein 4) [EC:3.4.16.4 3.4.21.-] D-alanyl-D-alanine carboxypeptidase (Penicillin-binding protein) family protein	FTN_0917 (K07259) Serine-type D-Ala-D-Ala carboxypeptidase/endopeptidase (penicillin-binding protein 4) [EC:3.4.16.4 3.4.21.-] serine-type D-Ala-D-Ala carboxypeptidase
**PBP-4**	No homolog found	FTL_1509 D-alanyl-D-alanine carboxypeptidase/D-alanyl-D-alanine-endopeptidase	FTN_0635 (K07259) serine-type D-Ala-D-Ala carboxypeptidase/endopeptidase (penicillin-binding protein 4) [EC:3.4.16.4 3.4.21.-] serine-type D-Ala-D-Ala carboxypeptidase
**PBP-5/6**	FTT_1029 (K07258) **dacD,** Serine-type D-Ala-D-Ala carboxypeptidase (penicillin-binding protein 5/6) [EC:3.4.16.4] D-alanyl-D-alanine carboxypeptidase	FTL_1060 (K07258) serine-type D-Ala-D-Ala carboxypeptidase (penicillin-binding protein 5/6) [EC:3.4.16.4] | (GenBank) D-alanyl-D-alanine carboxypeptidase (penicillin-binding protein) family protein	FTN_0907 (K07258) serine-type D-Ala-D-Ala carboxypeptidase (penicillin-binding protein 5/6) [EC:3.4.16.4]
**Penicillin-binding protein activator (LpoB)**	FTT_1540c (K07337) penicillin-binding protein activator hypothetical protein	FTL_0571 (K07337) penicillin-binding protein activator, conserved hypothetical protein	FTN_1449 (K07337) penicillin-binding protein activator, conserved protein of unknown function

**Research Question:** What is the role of the multiple penicillin-binding proteins (PBPs) and the activator LpoB in beta-lactam resistance in *Francisella?*

*Francisella*, as a genus, is largely resistant to penicillins and monobactam antibiotics (Scheel et al., 1993; Ikaheimo et al., 2000). Yet the resistance to cephalosporins appears to be variable across the genus. One study reports *Francisella* strains (clinical samples mostly from the southeastern and southwestern areas of the United States) to be susceptible to third-generation cephalosporins such as cefotaxime, moxalactam (latamoxef), and ceftazidime (Baker et al., 1985), yet another study indicates largely the opposite when testing Biovar II strains of *F. tularensis holarctica* (Tomaso et al., 2005). The resistance to carbapenems also appears to be variable across strains tested (Tomaso et al., 2005; Hotta et al., 2013). These disparities between studies are well summarized by Caspar and Maurin (2017). Unfortunately, the strains comparison across studies is difficult as the nomenclature of *Francisella* has changed considerably over the years, and many strains are annotated only as *F. tularensis*. Other factors precluding direct comparison include different testing methods of AMR, such as non-CLSI standard minimum inhibitory concentration (MIC) testing (Heine et al., 2017) and E-strip testing and the use of multiple media with various supplementations.

Beta-lactamase enzymes generally are of two main groups: metallo-beta-lactamases and serine-beta-lactamases (*bla* genes) (Conlan and North, 1992). Analysis of the genome sequences reveals that most *Francisella* strains possessed a *blaA* (β-lactamase class A) gene and a gene for *ampG* (Biswas et al., 2008). Amber class A β-lactamases are the most common beta-lactamase class found in bacteria resistant to β-lactam antibiotics. Metallo-β-lactamase family genes have not been identified in the *Francisella* genomes (Biswas et al., 2008). The known mechanisms of AMR to beta-lactams in *F. tularensis* revolve around two genes FTT_0681c and FTT_0611c, also known as bla1 and bla2 (or FTU-1) (Bina et al., 2006; Antunes et al., 2012). Bla2 is reported to be a non-carbapenem-hydrolyzing beta-lactamase which appears to be intrinsic in the genus and appears to confer a narrow-spectrum of resistance to beta-lactams, limited mainly to penicillins. This enzyme is classified as an Ambler Class A and Bush class 2f beta-lactamase (Juan et al., 2017).

The *F. tularensis holarctica* LVS equivalent gene of bla2 FTT_0611c (FTL_0879) was shown to be a functional beta-lactamase as its expression in *E. coli* increased the resistance of *E. coli* to penicillins, but not to third generation cephalosporins (Bina et al., 2006); thus, it is unlikely to be an extended-spectrum beta-lactamases (EBSL). Expression of FTT_0681c (FTL_0957) did not have the same effect. It was later shown that the protein product of FTT_0611c also acts as a very weak carbapenemase (Antunes et al., 2012; see section “Carbapenem Resistance”). Some *Francisella* strains also encode a third putative beta-lactamase gene FTT_0783, about which little is known beyond the fact that in LVS, FTL_1439 (equivalent to FTT_0783) appears to be more expressed at 26°C than 37°C, as judged by copy number (Cross and Jacobs, 1993; Horzempa et al., 2008). There may be additional mechanisms involved in beta-lactam resistance beyond these beta-lactamase enzymes such as penicillin-binding proteins or changes in cell permeability.

AmpG proteins are peptide-glycan specific permeases that are membrane proteins and can transport drugs through the same pathway used by murein components (Biswas et al., 2008). They are also annotated as major facilitator superfamily (MFS) proteins. In *E. coli*, AmpG transduces the signal that induces expression of the AmpC beta-lactamase protein in response to beta-lactams. Thus, AmpG in *E. coli* acts as a permease in peptidoglycan recycling and in the beta-lactamase induction system (Lindquist et al., 1993; Schmidt et al., 1995). The role of AmpG in *Francisella* has not been studied, nor the inducibility of the beta-lactamases. In *Francisella*, one *ampG* homolog gene has been identified in each of the major species (Table 3). Other components of this system in *E. coli* include AmpC, AmpR, AmpD, and AmpE. Of these, only AmpD (*N*-acetylmuramoyl-L-alanine amidase) has been annotated in *F. novicida* (FTN_1551) and *F. tularensis* (FTT_0162).

**TABLE 3:** Putative AmpG permease homologs (MFS protein) in *Francisella* (selected genes from Uniprot.org).

Locus and gene names	Organism	Uniprot entry	Protein names	Length
**FTN_1641 (AmpG)**	*Francisella tularensis* subsp. *novicida* (strain U112)	A0Q8D4	Peptide-acetyl-coenzyme A transporter (PAT) family protein	421
**FTL_1790**	*Francisella tularensis* subsp. *holarctica* LVS	A0A0B3WLC8	AmpG family muropeptide MFS transporter	421
**FTT_0070c (AmpG)**	*Francisella tularensis* subsp. *tularensis* (strain SCHU S4)	Q5NIJ7	Major facilitator superfamily (MFS) transport protein	421

**Research Question:** Is *ampG* expression inducible by beta-lactams and what is its role in beta-lactam resistance in *Francisella?*

### Monobactam Resistance

Monobactams are specific class of beta-lactams that have beta-lactam rings that are not fused to other rings, unlike most other beta-lactams such as penicillin. They are resistant to enzymatic degradation by metallo-beta-lactamases, but are generally susceptible to degradation by serine beta-lactamases. These antibiotics are generally effective against aerobic, Gram-negative bacteria. The most common example of this class is Aztreonam. *Francisella* is generally considered to be resistant to monobactams through the same beta-lactamase genes as confer beta-lactam resistance (Caspar and Maurin, 2017). Resistance to aztreonam is reported in *F. tularensis holarctica* strains (Scheel et al., 1993; Garcia del Blanco et al., 2004; Tomaso et al., 2005). For Japanese strains of *F. tularensis* subsp. *holarctica biovar japonica*, the MIC is reported to range from 0.75 to >256 μg/mL aztreonam (Hotta et al., 2020). Interestingly, it was found that aztreonam demonstrated a significant inhibition of biofilm formation for *F. novicida* (Dean and van Hoek, 2015). Recently, the class A β-lactamase gene FTU-1 in *F. tularensis* (see section “Carbapenem Resistance”) was found to be unable to hydrolyze aztreonam (Antunes et al., 2012).

### Carbapenem Resistance

Carbapenems are also members of the beta-lactam class of antibiotics. They consist of the classic beta-lactam ring fused at the nitrogen and a neighboring carbon to an additional ringed three carbons. Regarding the carbon immediately neighboring the nitrogen along the additional ring, there is a double bond (to the adjoining carbon) and a carboxylic acid. All other positions are variable. Due to this structure, carbapenems differ from beta-lactams such as penicillin in that they are not typically cleaved by beta-lactamases. *Francisella* as a genus has mixed resistance to carbapenem antibiotics (Caspar and Maurin, 2017) which is thought to be conferred by the Class A carbapenemase FPH-1 in *Francisella philomiragia* and potentially the *F. tularensis blaA2* gene (Bina et al., 2006). The European holarctic strains are all resistant to imipenem, and several strains are resistant to meropenem, with Japanese strains showing mixed sensitivity/resistance to imipenem and carbapenem (Caspar and Maurin, 2017).

Carbapenem-resistance in *E. coli* and other Gram-negative bacteria is often mediated by the acquisition of mobile genetic elements that confer this phenotype. *Francisella* appears to have some inherent carbapenem resistance via the beta-lactamase genes, which are chromosomally located not plasmid-mediated. *F. philomiragia* is reported to have a chromosomally encoded Class A carbapenemase gene, FPH-1 (Toth et al., 2012). Class A carbapenemases are reported to confer reduced susceptibility to imipenem to bacteria expressing them (Naas et al., 2016). FPH-1 appears to provide a broad spectrum of resistance, including expanded-spectrum cephalosporins, aztreonam, and carbapenems. The *Francisella blaA2* chromosomal gene FTT0611c which encodes FTU-1 has been reported to act as a very weak carbapenemase (Antunes et al., 2012). In published experiments, FTU-1 expression elevates the MIC of only imipenem twofold (Antunes et al., 2012), while FPH-1 elevates the MICs (8x–64x) of imipenem, meropenem, ertapenem, and doripenem (Toth et al., 2012). Based on their somewhat unusual sequences and their activity profiles, these *Francisella* beta-lactamase genes (FPH-1, FTU-1, and FTU-2) have been placed on a new and distinct branch of the class A beta-lactamase tree (Naas et al., 2016).

### Aminoglycoside Resistance

While the reported *in vitro* MICs for many antibiotic classes (Maurin et al., 2000) are low against *Francisella*, clinical tularemia treatment involves only three antibiotic classes: the fluoroquinolones, tetracyclines, and aminoglycosides (Boisset et al., 2014; Caspar and Maurin, 2017). However, treatment failures with these classes of antibiotics have been reported for up to 25% of tularemia cases (Perez-Castrillon et al., 2001; Boisset et al., 2014; Caspar and Maurin, 2017). Aminoglycoside antibiotics include gentamicin, streptomycin, amikacin, and tobramycin. Of these, only streptomycin has demonstrated a 100 % cure rate for tularemia, but is not commonly used due to the intramuscular administration route and potential toxicity (Foshay and Pasternack, 1946; Enderlin et al., 1994). More recently, the fluoroquinolones such as ciprofloxacin and levofloxacin (Levaquin) have been shown to be effective *in vitro* and *in vivo* against this infection (Klimpel et al., 2008; Nelson et al., 2010; Peterson et al., 2010; Propst et al., 2016a; Caspar and Maurin, 2017). Assessment of the susceptibility of intracellular bacteria to these antibiotics may more closely mimic the *in vivo* response to these antibiotics (Maurin et al., 2000). Despite treatment failures, naturally occurring *Francisella* strains with aminoglycoside resistance have not been reported. Multiple transposon mutants in *F. novicida* have been identified as being hypersensitive to both gentamicin and spectromycin (see Figure 3 in Enstrom et al., 2012), including mutatations in *hslVU*, *yccA*, and *sohB*, all genes associated with proteolysis.

Spectinomycin is an antibiotic of the aminocyclitol class of antibiotics which inhibits protein synthesis by reversibly binding the 16S RNA of the 30S ribosomal subunit, and has a bacteriostatic mode of action (Chopra, 2010). It has been used to treat gonorrhea infections. New derivatives of spectinomycin (novel *N*-benzyl aminomethyl spectinomycins) were found to be effective *in vivo* in a murine model against *F. tularensis* (Scarff et al., 2019). A low incidence of spontaneous AMR to spectinomycin has been reported in *Francisella* (Kormilitsyna and Marakusha, 1983), but in the laboratory, resistant mutants were readily obtained *in vitro* and under selection pressure (Bhatnagar et al., 1994). Thus, this antibiotic demonstrates potential for use, especially against a fluorquinolone antibiotic-resistant strain, if one were to be identified clinically.

Aminoglycoside resistance in *Francisella* is an interesting topic. While there are known aminoglycoside-modifying proteins in other genera, none are known in *Francisella*. The known mechanisms of resistance to aminoglycosides in *Francisella* are efflux-pump-based (Table 4).

**TABLE 4:** The annotated efflux pumps in *Francisella* species and subspecies.

	*Francisella tularensis* subsp. *tularensis* Schu S4	*Francisella tularensis* subsp. *holarctica* LVS	*Francisella tularensis* subsp. *novicida* U112
	Locus (KEGG orthology, Uniprot) name [E.C. number], other information	Locus (KEGG orthology, Uniprot) name [E.C. number], other information	Locus (KEGG orthology, Uniprot) name [E.C. number], other information

**Outer membrane efflux proteins**			

**TolC** (OMP), outer membrane efflux protein, tolC precursor	FTT_1724c (K12340)	FTL_1865 (K12340)	FTN_1703 (K12340, A0Q8J5)

**AcrAB system**			

**AcrA**, efflux transporter, RND family, MFP subunit. MexH family. (Also annotated as a Co/Zn/Cd efflux system membrane fusion protein in some strains)	FTT_0106c (K03585)	FTL_1671 (K03585)	FTN_1609 (K03585, A0Q8A4), RND family efflux transporter, MFP subunit. MexAB-OprM [MD:M00718]
**AcrB** (RND)	FTT_0105c transporter AcrB/AcrD/AcrF family	FTL_1672 (Bina et al., 2008)	FTN_1610 (K18138, A0Q8A5) AcrB/AcrD/AcrF family
**FtlC**	FTT_1095c	FTL_1107	FTN_0779

**Emr MFS-type multidrug efflux pump (EMR locus) (Alqahtani et al., 2018)**			

**SilC**	FTT_1258	FTL_0686 (Alqahtani et al., 2018)	FTN_1277
**EmrA1**, outer membrane efflux protein,	FTT_1257 (K03543)	FTL_0687 (K03543) (Alqahtani et al., 2018)	FTN_1276 (K03543)
**EmrB**, membrane fusion protein, multidrug efflux system membrane fusion protein; membrane fusion component of tripartite multidrug resistance system	FTT_1256 (K03446)	FTL_0688 (K03446) (Alqahtani et al., 2018)	FTN_1275 (K03446)

**Other efflux systems**			

Efflux transporter, RND family, MFP subunit.	FTT_0747c, hypothetical protein (261 aa), HlyD_D23 domain	FTL_1366 (partial, 105 aa)	FTN_0718 (Uniprot A0Q5U5), (285 aa), membrane fusion protein
**ErmE** multidrug resistance antiporter of cations and cationic drugs, small multidrug resistance family proteins			FTN_0799
**ArsB** (arsenite-antimonite efflux family protein)	FTT_0853	Not found	FTN_0382 (A0Q4X0) FTN_0800
**ArsR** family transcriptional regulator K03892, arsenate/arsenite/antimonite-responsive transcriptional repressor (Xu et al., 1996)	FTT_0868c	FTL_0370	FTN_0395 FTN_0801

**FIGURE 1:**
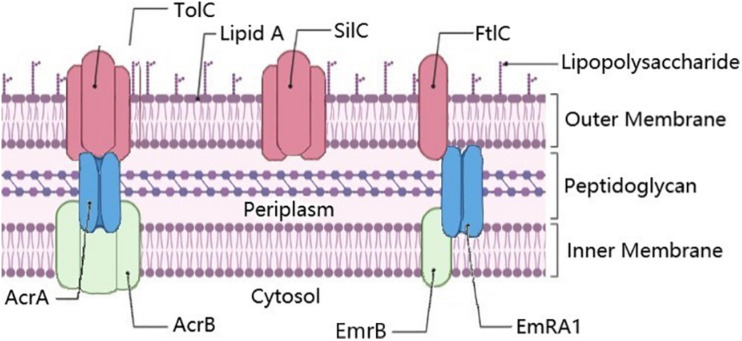
Annotated efflux pump genes in *Francisella*. The membrane fusion proteins AcrA and EmrA1 proteins are shown in blue, the plasma membrane fusion proteins AcrB and EmrB in Green, and outer membrane proteins TolC, SilC, and FtlC in pink. SilC may also pair with EmrA1 and EmrB, especially as they are co-localized in the chromosome. Known trimers illustrated as such. Figure created using BioRender (https://biorender.com/).

The chromosome of *Francisella* bacteria encodes several multidrug efflux pumps (Table 4). AcrAB complex operates in conjunction with an outer membrane porin (such as TolC) to pump various substrates out of the cell as a multidrug efflux pump (Bina et al., 2008) (Figure 1). AcrA (FTT_0106c) is a cytoplasmic-membrane fusion protein, while AcrB (FTT_0105c) is an efflux pump protein. AcrAB may be involved in beta-lactam and cephalosporin resistance (Bina et al., 2008; Caspar and Maurin, 2017); for example, AcrAB was found to be important for azithromycin resistance (Ahmad et al., 2010). AcrAB is also responsible for some detergent resistance in *Francisella.* Interestingly, when combined with non-ionic detergents, cephalosporins appear to be more effective against *Francisella*, whereas the same is not true of ampicillin, suggesting differential mechanisms of resistance (Pavlovich and Tsimbalistova, 2019), while simultaneously suggesting a potential synergistic mechanism based around the overloading of AcrAB.

In LVS, the outer membrane porin TolC (FTL_1865) (Table 4) was shown to be involved with resistance to gentamicin (Gil et al., 2006). Gentamicin is a common drug for treating patients with tularemia, which targets the 30s ribosome and leads to inhibition of protein synthesis (Loughman et al., 2016). FtlC (FTL_1107), a TolC-like protein, is partially responsible for the AMR of *Francisella* against kanamycin and streptomycin FtlC (FTL_1107) (Gil et al., 2006). A third TolC-like protein encoding gene called SilC (FTL_0686), adjoining *EmrA1/B* a cytoplasmic membrane fusion protein gene (FTL_0687)/efflux pump pair (FTL_0688), is also found in many *Francisella* strains, including Schu S4 (Table 4) (Alqahtani et al., 2018). EmrB in *E. coli* is a known multiple drug resistance efflux pump (Lomovskaya and Lewis, 1992), along with EmrA1. It appears that EmrA1 is involved with resistance to streptomycin and neomycin but not gentamicin (Ma et al., 2014), as well as SDS (Enstrom et al., 2012) in *Francisella*. The relationship and interchangeability of the various TolC orthologs in Francisella are more thoroughly discussed in Kopping et al. (2019). The various pumps/membrane proteins/fusion proteins in *Francisella* are illustrated in Figure 1.

**Research Question:** What is the link between AcrAB, non-ionic detergents, and beta-lactam resistance in *Francisella?*

Interestingly, gentamicin resistance in Francisella can be modulated by temperature. This was demonstrated via gentamicin conjugated to fluorescent dye entering the cell at different rates, with diminished antibiotic uptake at 26°C than 37°C, perhaps suggesting that *Francisella* in environmental conditions as opposed to host conditions may acquire phenotypic resistance to compounds produced by other soil microbes, such as gentamicin (Loughman et al., 2016). The mechanism of this interesting phenotypic resistance mechanism has yet to be demonstrated, and is perhaps linked to the as-yet-unknown PMF-powered mechanism of gentamicin uptake into *Francisella*.

### Tetracycline Resistance

Tetracycline is bacteriostatic for *Francisella*, and treatment of tularemia with this antibiotic can lead to relapses if treatment is not given for at least 14 days (Enderlin et al., 1994; Maurin et al., 2000; Perez-Castrillon et al., 2001). Most *Francisella* strains are susceptible to tetracycline *in vitro* with MIC ranging from 2.0 to 0.094 μg/mL (Baker et al., 1985; Ikaheimo et al., 2000). However, the clinical response of tularemia patients to the bacteriostatic antibiotic tetracycline appears to be subject to relapses and treatment failure (Baker et al., 1985; Maurin et al., 2000). Research on potential tetracycline resistance in *Francisella* is scant. A few studies have suggested that efflux pumps might be a potential mechanism of developing tetracycline resistance. Both TolC (Gil et al., 2006) and EmrA1 (Ma et al., 2014) proteins were also indicated in *Francisella’*s resistance to tetracycline *in vitro* (Table 4). Tetracycline resistance has been engineered via plasmid into *Francisella* (Pavlov et al., 1996; LoVullo et al., 2012; McWhinnie and Nano, 2014), demonstrating that this resistance is certainly possible by introducing a tetracycline pump as a selection marker. In addition, a plasmid was constructed for *Francisella* to enable tetracycline-inducible protein-expression system in this organism (Sheshko et al., 2021).

Interestingly, bioinformatics analysis revealed the presence of tetracycline resistance protein (Tet) in most strains of *Francisella* (Biswas et al., 2008). Shown in Table 5 are the identified or annotated Tet protein genes in *Francisella*. This protein is an MFS-type multidrug transporter, which suggests that *Francisella* could pump out this compound as a mechanism of resistance.

**TABLE 5:** Identification of putative tetracycline resistance genes (Tet) in most strains of *Francisella* (selected data from Uniprot.org).

Gene locus number	Organism	UniProt entry	NCBI-ProteinID	Protein names	Protein length
**FTT_0444 (*tet*)**	*Francisella tularensis* subsp. *tularensis* (strain SCHU S4/Schu 4)	Q5NHK9	YP_169483	Multidrug transporter MFS_1 (tetracycline resistance protein)	412
**FTL_1622**	*Francisella tularensis* subsp. *holarctica* LVS	A0A0B3VY25	CAJ80061	MFS transporter (Sugar (And other) transporter family protein)	408
**FTN_0535**	*Francisella tularensis* subsp. *novicida* strain U112	A0Q5B5	ABK89430	Drug:H+ antiporter-1 (DHA1) family protein	408

Doxycycline, also a member of the tetracycline class, is among the drugs recommended by the CDC for treatment of tularemia (Dennis et al., 2001) and is bacteriostatic. Generally, doxycycline is very effective against tularemia with an MIC reported between 0.064 and 4 mg/L and no doxycycline-resistant strains have been reported in nature (Caspar and Maurin, 2017). Interestingly, ciprofloxacin-resistant *Francisella* strains have an approximately 2–10-fold increase in the MIC for doxycycline in two studies, suggesting that some cross-resistance may be happening (Biot et al., 2020; Sutera et al., 2014b). There is overall very little research on the mechanism and frequency of doxycycline resistance in *Francisella*.

**Research Question:** What is the role of tetracycline resistance protein (Tet) in tetracycline sensitivity or resistance in *Francisella?*

### Polymyxin Resistance

Polymyxins are cationic cyclic peptide antibiotics that are drugs of last resort for many multidrug resistant infections. They are also often invoked as models for the mechanism of action of innate immune system cationic antimicrobial peptides. Polymyxin resistance is usually due to modification of the LPS of Gram-negative bacteria (Schaenzer and Wright, 2020), in particular the Lipid A (Li et al., 2012). *Francisella* is also known to be highly resistant to polymyxins (Llewellyn et al., 2012; Stephens et al., 2016), and it is used as part of *Francisella* selective media to counter select against faster growing organisms. In fact, 100 μg/mL polymyxin B is added to selective media for the isolation of *Francisella* from organ homogenates (Petersen et al., 2004a, b, 2009). *Francisella* LPS varies by strain, but its LPS is unusual as compared to that of other Gram-negative bacteria as it does not activate Toll-like receptor 4, a host protein largely responsible for inflammation in response to bacterial LPS (endotoxin) (Gunn and Ernst, 2007). Specifically, the *Francisella* LPS is tetra-acylated and has C16–C18 length tails, which is quite different than *E. coli* LPS (Vinogradov et al., 2002; Li et al., 2012; Okan and Kasper, 2013).

The main mechanism of resistance is through polymyxin binding of LPS, and most of the “Polymyxin Resistance” genes annotated in *Francisella* (Table 6) are involved with either the assembly or creation of LPS components (Sampson et al., 2014). The lipid A component in *Francisella* is different from that of *E. coli* in that the lipid A of *Francisella* has the hydroxyl group of the 2’-linked fatty acyl chain of lipid A esterified with C16:0. In contrast, *E. coli* has two phosphates at the 1’ and 4’. *Francisella* lipid A also has an unusual α-linked galactosamine addition at the 1-position and is lacking the traditional phosphate at the 4’-position of the carbon ring (Okan and Kasper, 2013). There are a handful of proteins known to enable these LPS modifications. The role of some of these genes including *lpxD1* and *lpxD2* (Li et al., 2012) as well as *lpxF* (Wang et al., 2007) in polymyxin resistance was confirmed experimentally in *Francisella*. LpxD1 and LpxD2 are responsible for acylating LPS (Yun et al., 2017), whereas LpxF is responsible for dephosphorylation of 4’ phosphates in *Francisella* LPS (Wang et al., 2006). A deletion mutant in *F. novicida* LpxD2 conferred significant increase in polymyxin B resistance, while a deletion mutant in *lpxD1* caused a sensitivity to polymyxin B (Li et al., 2012). Indeed, the *lpxD1*-Null Mutant is both avirulent and protective as an attenuated vaccine strain (Li et al., 2012). LpxA, a protein which catalyzes lipid A synthesis (Joo and Chung, 2016), from *F. novicida* was shown to be active in the production of Lipid A when expressed in *E. coli* (Joo and Chung, 2016).

**TABLE 6:** Putative polymyxin resistance genes in *Francisella* (selected data from Uniprot.org and Kegg.jp).

Locus (gene) names	Organism	UniProt entry	Phenotype	Protein names	Length	References
**LpxF type**						

**FTN_0295 (*lpxF* )**	*Francisella tularensis* subsp. *novicida* (strain U112)	A0Q4N6	Polymyxin Sensitivity	Lipid A 4’-phosphatase	222	Wang et al., 2007
**FTT_1634c**	*Francisella tularensis* subsp. *tularensis* (strain SCHU S4)	Q5NEJ5		Hypothetical Protein	222	
**FTL_1704**	*Francisella tularensis* subsp. *holarctica* LVS	Q2A1R7		Hypothetical Membrane Protein	222	

**ArnT type**						

**FN3523_1260**	*Francisella hispaniensis*	AEE26563		**Polymyxin resistance protein ArnT**, undecaprenyl phosphate-alpha-L-Ara4N transferase Melittin resistance protein PqaB	587	
**FTT_0455c**	*Francisella tularensis* subsp. *tularensis* SCHU S4	Q5NHJ9 (YP_169493)		Dolichyl-phosphate-mannose-protein mannosyltransferase family protein	587	Phillips et al., 2004
**FTN_0546**	*Francisella tularensis* subsp. *novicida* (strain U112)	A0Q5C6		Dolichyl-phosphate-mannose-protein mannosyltransferase family protein	587	
**FTL_1609**	*Francisella tularensis* subsp. *holarctica* LVS	CAJ80048		Dolichyl-phosphate-mannose-protein mannosyltransferase family protein	586	

**ArnC Type**						

**FN3523_1261**	*Francisella hispaniensis*	F4BGH0		**Polymyxin resistance protein ArnC,** glycosyl transferase	317	
**FTT_0454 (yfdH)**	*Francisella tularensis* subsp. *tularensis* (strain SCHU S4)	Q5NHK0		Glycosyl transferase, group 2 family protein	318	
**FTN_0545**	*Francisella tularensis* subsp. *novicida* (strain U112)	A0Q5C5		Glycosyl transferase, group 2	318	
**FTL_1611**	*Francisella tularensis* subsp. *holarctica* LVS	A0A0B6E7W3 (CAJ80050)		Glycosyl transferase, group 2 family protein	317	

*At least three categories of Francisella genes have “polymyxin resistance” terms associated with them: lpxF, arnC, and arnT genes.*

Polymyxin resistance also involves bacterial lipoprotein (BLP) regulated by the CRISPR-Cas protein Cas9 in *F. novicida* (Sampson et al., 2013). Repression of BLP expression leads to enhanced polymyxin B resistance, suggesting that this protein may play a role in the action of polymyxin as an antibiotic in *Francisella*.

Another pair of genes, including *arnT* (undecaprenyl phosphate-alpha-L-Ara4N transferase) and *arnC* (glycosyl transferase family 2, FTU_0505), are also annotated as “Polymyxin resistance genes” in some *Francisella* strains and have homologs in other strains (Table 6) (Li et al., 2012). ArnC and ArnT add sugars to LPS following the dephosphorylation event performed by LpxD1 and LpxD2 (Okan and Kasper, 2013). The nature of these additions is strain dependent and the role, if any, of these genes in Francisella antibiotic resistance has not been determined.

In some cases, *Francisella*’s resistance to polymyxin can also be affected by other signaling pathways, including the two-component system which can alter the expression of multiple genes, including those that affect polymyxin resistance. We found that the *Francisella* two-component system response regulator BfpR (FTN_1452/FTT_1543) regulates the expression of a gene encoding glycosyltransferase potentially similar to *arnC* (FTN_0545, FlmF2, or yfdH) (Richards et al., 2008; Wang et al., 2009), which was found to be significantly deceased (0.45-fold) in the *bfpR* mutant strain relative to wild-type *F. novicida* (Dean et al., 2020).

### Cationic Antimicrobial Peptide Resistance

Gram-negative bacteria have multiple known mechanisms by which they can resist the action of the cationic antimicrobial peptides (Band and Weiss, 2015), which are being investigated as antibacterial agents against *Francisella* (Han et al., 2008; Amer et al., 2010; Flick-Smith et al., 2013; Chung et al., 2015; Findlay et al., 2016; Kaushal et al., 2016; Propst et al., 2016a; Dean et al., 2020). Polymyxin B and colistin are both cationic polypeptide antibiotics. KEGG has annotated the following genes as playing a role in cationic antimicrobial peptide resistance in *Francisella* (KEGG, 2020): The multidrug resistance efflux pump of the MexAB-OprM class *tolC*, *acrA*, *acrB* (FTT_1742c [FTN_1703], FTT_1016c [FTN_1609], and FTT_0105c [FTN_1610], respectively) and *lpxA*, an LPS biosynthesis gene (FTT_1569c [FTN1478]). However, this annotation as being important in cationic antimicrobial peptide resistance has not been experimentally confirmed for any of these. *tolC* mutants had the same polymyxin sensitivity as wild-type *F. tularensis* SchuS4 (Kopping et al., 2019). Experiments examining the importance of AcrA/B/TolC in *F. tularensis LVS* have also not demonstrated a role for these genes in polymyxin resistance (Gil et al., 2006; Kopping et al., 2019). *LpxA*, the LPS biosynthesis gene FTT_1569c [FTN1478], was annotated by KEGG as playing a role in cationic antimicrobial peptide resistance and interacting with AcrA/B/TolC. Resistance to the cationic polypeptide polymyxin in *Francisella* is thought to be mediated mainly by its LPS as discussed above.

Recently, we have demonstrated that mutants in the two-component system BfpR in *Francisella* lead to a phenotype of antimicrobial peptide resistance in *F. novicida* for two antimicrobial peptides, the human cathelicidin peptide LL-37 and a sheep antimicrobial peptide SMAP-29 (Dean et al., 2020). This mechanism may be through regulation of biofilm formation, or some yet undetermined mechanism.

**Research Question:** What are the genes responsible for resistance to cationic antimicrobial peptides in *Francisella?* Are there any other genes in addition to LPS synthesis genes that play a role?

### Macrolide Resistance (Erythromycin) and Sensitivity (Azithromycin)

Macrolides are polyketide antibiotics that are typically bacteriostatic and work by inhibiting protein synthesis through binding to the 50S ribosome. Macrolides include erythromycin and azithromycin.

Erythromycin has limited efficacy against many Gram-negative bacteria due to its hydrophobic nature and lack of permeability through the Gram-negative outer membrane (Saha et al., 2008). Some strains of *Francisella* are intrinsically resistant to erythromycin; no reports of acquired resistance have been found. Interestingly, different sub-strains of *Francisella* have different susceptibility to erythromycin. The Eurasian strains (biovar II) of *Francisella* are often erythromycin resistant (Urich and Petersen, 2008; Karlsson et al., 2016). In the *holarctica* strains (*F. tularensis* subsp. *holarctica*), biovar I was found to be erythromycin sensitive, whereas biovar II B.12 strains including the live vaccine strain (LVS) were found to be erythromycin-resistant (Kudelina and Olsufiev, 1980). A common mechanism of resistance to macrolides is the modification of the 23S rRNA, the *rrl* gene. *F. tularensis* LVS was found to have a point mutation in Domain V of the 23S rRNA, rendering it more resistant to erythromycin than *F. novicida* or *F. tularensis* Schu S4 (Biswas et al., 2008). This modification could explain the increased resistance to erythromycin in *F. tularensis* LVS. SNP analysis confirmed that this resistance was due to the presence of A→C SNP at position 2059 in the three copies of the *rrl* gene. Introducing this mutation into erythromycin-sensitive *Francisella* strains rendered them resistant (Karlsson et al., 2016). Exposure *in vitro* of *F. tularensis* subsp. *holarctica* biovar I strains to increasing erythromycin concentrations can lead to resistance mutations in 23S RNA and other sites as well as upregulation of efflux pumps (Gestin et al., 2010). In the North American Type A *Francisella* strains, erythromycin MICs range from 0.5 to 2 μg/mL, while *F. tularensis holarctic* strains have observed MICs of 4 to >256 μg/mL (Marinov et al., 2009; Caspar and Maurin, 2017).

In addition, certain methylases can confer increased resistance by methylation of a specific adenine residue of the 23S rRNA. Methylases that have been identified as *Francisella* critical virulence factors might have this activity (Kraemer et al., 2009). Some methylases present in the genome of *F. novicida* are either absent or pseudogenes/non-functional genes in *F. tularensis* Schu S4 (such as FTT0010, FTT0770, FTT1430, FTT1719, and FTT1735c), potentially contributing to the different sensitivities to erythromycin between the strains (Larsson et al., 2005).

**Research Question:** What is the potential role of these methylases in acquired erythromycin resistance in *Francisella?* This has not yet been experimentally determined.

Azithromycin (Zithromax) is a very commonly prescribed macrolide antibiotic for the pediatric populations. Therefore, there is interest in its potential use for treating pediatric tularemia patients (CIDRAP, 2013). Azithromycin is currently approved as a pediatric macrolide in use in the United States and Europe. Azithromycin has the amazing pharmacokinetic property of concentrating inside macrophages up to 1000x the serum concentration. We found that azithromycin was more effective against the Gram-negative *Francisella* bacteria than was expected (Ahmad et al., 2010). Despite reports that European clinical strains of Type B *F*. *tularensis holarctica* and Japanese *palaearctica* biovar strains are resistant to azithromycin *in vitro* (MIC > 256 μg/mL) (Ikaheimo et al., 2000), we observed that commonly used laboratory strains were sensitive to this antibiotic. We have demonstrated that the Type A *F. tularensis tularensis* strains are sensitive to azithromycin *in vitro* (Ahmad et al., 2010). *F. philomiragia* (an environmental strain of *Francisella*) and *F. novicida* are also sensitive to this drug with similar MICs. The MIC for *F. tularensis* LVS (NR-646) was 25 μg/mL azithromycin, confirming the finding that LVS and *holarctic* strains are relatively more resistant than other *Francisella* strains. Furthermore, due to the high concentrating effect in macrophages for azithromycin (approximately ∼1000x the serum concentration), the bacteria were effectively killed in the intracellular state by this antibiotic (Ahmad et al., 2010). As mentioned above, AcrAB, the inner-membrane component of the TolC/AcrAB Type 1 secretion system, was found to be important for azithromycin resistance (Ahmad et al., 2010).

### Rifampin Resistance

Rifampin, also known as Rifampicin, is a broad-spectrum antibiotic used to treat bacterial infections, mostly tuberculosis in humans. In veterinary medicine, rifampin is used to treat various Gram-positive infections including *Staphylococcus*, *Streptococcus*, and most strains of *Bacteroides*, *Clostridium*, *Neisseria*, and *Listeria*. Gram-negative organisms are not usually affected by rifampin at the typical doses administered. Rifampin works by inhibiting transcription, specifically by interfering with bacterial DNA-dependent RNA polymerase (Chen and Kaye, 2011; Alifano et al., 2015). Rifampin has a very interesting pharmacokinetic profile, which includes accumulating inside cells and thus can exert antibacterial effects against intracellular bacteria (Chen and Kaye, 2011).

Resistance to rifampin in *E. coli* is due to mutations in the *rpoB* gene, which affects the activity of RNA polymerase (Chen and Kaye, 2011). This mutation has not been seen clinically for *Francisella*. Experimentally, rifampin resistance has been generated by growth under the increasing concentration of rifampin. When these mutants were tested for their virulence *in vivo*, it was found that these mutants had decreased virulence compared to the wild-type *Francisella* (Bhatnagar et al., 1994). There is one transposon-insertion mutant available in *rpoB* in *F. novicida* (Gallagher et al., 2007), suggesting that this gene is not an absolutely essential gene.

An emerging use for rifampin is as an adjunct therapy or combination therapy with other antibiotics or antimicrobials in other organisms (Maslow and Portal-Celhay, 2015). Rifampin inhibition of *Francisella* growth is bacteriostatic (Baker et al., 1985), but it could be used in combination with other antibiotics or possibly in combination with antimicrobial peptides, as we recently demonstrated for *Mycobacteria* (Gupta et al., 2015). The synergy between colistin and rifampin has been observed in several multidrug-resistant bacteria, further supporting this approach (Chen and Kaye, 2011).

### Fosmidomycin Resistance and the DXR/MEP Pathway

Bacteria, algae, and plants all produce isoprenoids through the methylerythritol phosphate (MEP) pathway. As the MEP pathway is only present in prokaryotes and some lower eukaryotes, but not in mammalian cells, the pathway is attractive for antimicrobial drug development (Jawaid et al., 2009; Mackie et al., 2012; McKenney et al., 2012). Genes in the MEP pathway have been identified in many bacterial biothreat agents, including *Francisella*. The importance of the MEP pathway to *Francisella* is illustrated by the lethality of mutations in the MEP pathway genes (Jawaid et al., 2009; McKenney et al., 2012).

**FIGURE 2:**
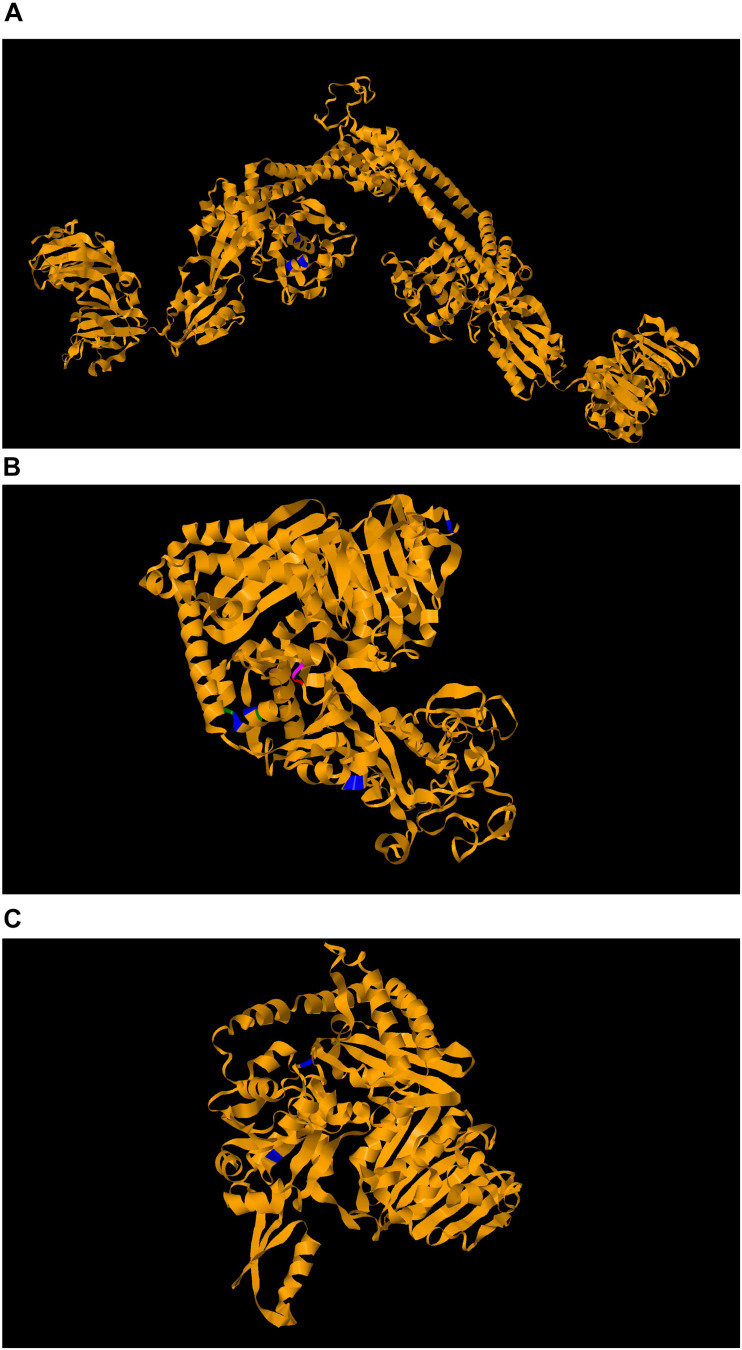
DNA gyrase in *Francisella*, showing critical residues. The *Francisella tularensis tularensis* ScuS4 GyrA **(A)**, GyrB **(B)**, and ParE **(C)** amino acid sequences were used in conjugation with SwissModel (Schwede et al., 2003) to generate three-dimensional protein models. The model of each with the highest global model quality estimation score is presented here in orange. On each model, mutations leading to enhanced resistance were mapped. Blue indicates a substitution of residue, red a deletion, green an insertion after the indicated residue, and magenta either a deletion or a substitution at that residue. Protein models were built in SWISS-MODEL and illustrated in Geneious (Schwede et al., 2003; Kearse et al., 2012).

The first committed step of the MEP pathway is catalyzed by the enzyme 1-deoxy-D-xylulose 5-phosphate reductoisomerase (DXR/MEP synthase) (Jawaid et al., 2009). We have previously established that fosmidomycin and the prodrug FR900098 inhibit purified *F. tularensis* LVS MEP synthase enzyme (DXR IC50 = 230 nM) (Jawaid et al., 2009). We subsequently demonstrated that fosmidomycin is somewhat effective against the bacteria with an MIC of 136 μM, and strongly suppressed the growth of intracellular *Francisella* suggesting that fosmidomycin and the prodrug may be active *in vitro* against this organism (McKenney et al., 2012). We cultured *F. novicida* with FR900098 antibiotic disks and selected the resistant mutants that appeared for sequencing. Resistance to fosmidomycin was not directly developed in the *DXR* gene, as one might predict, but rather turned out to be an indirect target, through mutation of a transporter, GlpT (Mackie et al., 2012). This transporter is responsible for the ability of the drug to enter the bacteria, and mutations in the transporter render the antibiotic ineffective as demonstrated by colonies appearing in the inhibition zone. This transporter mutant turned out to be a very powerful screen for lipophilic versions of fosmidomycin-related compounds that can act on intracellular *Francisella* and DXR in a GlpT-independent manner (Figure 2).

### Emerging Fluoroquinolone Resistance in *Francisella*

Fluoroquinolones are broad-spectrum antibiotics which inactivate bacterial topoisomerases and gyrases, which are responsible for the supercoiling of bacterial DNA and, therefore, its replication. Many proposed mechanisms of resistance to fluoroquinolones in *Francisella* revolve around the mutation of the antibiotic target. As shown in serial passage experiments in *Francisella*, mutations in type II gyrases GyrA (FTT_1575c) and GyrB (FTT_0510), as well as mutations in type IV topoisomerases ParE (FTT_0163c) or ParC (FTT_0396), confer enhanced resistance to fluoroquinolones (Sutera et al., 2014b) (Figure 2). A similar study in *Francisella* LVS also identified GyrA as the main target of induced ciprofloxacin resistance (Jaing et al., 2016). The specific regions altered in Gyr A that give *Francisella* enhanced resistance are residues 83 and 87, which are involved in the catalytic domain, 43, 523, and 524 which are involved with DNA binding (Figure 2A). Those altered in GyrB are catalytic residues 464–466, DNA binding residues 486, 487, and 747, as well as mutation in residues 570 and 86, which have unknown roles in protein functionality (Sutera et al., 2014b; Caspar et al., 2017) (Figure 2B).

The mutations on ParEC followed a similar pattern with the catalytic residue 82 of ParC and the DNA binding residue of ParE 472 (Sutera et al., 2014b) (Figure 2C). However, these mutations alone could not explain differential MIC results, suggesting that there may be secondary mechanisms which contribute to fluoroquinolone resistance. This disparity is further highlighted by the fact that tularemia patients who failed to respond to fluoroquinolone treatment did not contain *Francisella* with gyrase and topoisomerase mutations (Sutera et al., 2017). This suggests that alternative mechanisms of resistance, such as efflux pumps, which may also contribute to fluoroquinolone resistance, may be important as well (Sutera et al., 2014b). Efflux pump components TolC (Gil et al., 2006), EmrA1 (Ma et al., 2014), and SilC (Alqahtani et al., 2018) have been associated with resistance to the quinolone nalidixic acid, supporting this hypothesis.

It was found that deletion of *FupA/B* gene (FTT_0918), which encodes an iron-binding membrane protein, also contributed to fluoroquinolone resistance. While the exact mechanism is unclear, the authors suggest that deletion of FupA/B destabilizes the cell membrane leading to a larger production of OMVs, which may shield the bacteria from the antibiotic in some way (Siebert et al., 2019).

Transposon insertions into *recABCD* and *ruvABC* genes increased the sensitivity to quinolones in *F. novicida*, and the replication helicase (*rep*) and exonuclease I (*sbcB*) may also play a role (Enstrom et al., 2012).

## Efflux Pumps in *Francisella*

*Francisella* species contain genes for very few efflux pumps (Table 4 and Figure 1). Overall, there are three closely related pumps, classified as Type I secretion systems, including the TolC/AcrA/B efflux pump (Kopping et al., 2019), the TolC homolog FtlC (Gil et al., 2006), as well as SilC and the HylD/EmrA1 system (Ma et al., 2014; Alqahtani et al., 2018). The TolC/AcrA/AcrB complex is an important and well-characterized multidrug-resistant efflux pump in *Francisella* (Kopping et al., 2019). As described in the sections above, the TolC/AcrA/AcrB efflux pump is shown to play important roles in erythromycin, SDS (Kopping et al., 2019), and aminoglycoside resistance (Gil et al., 2006). SilC/EmrA1 was shown to be important in streptomycin and polymyxin resistance (Alqahtani et al., 2018), although not polymyxin resistance in SchuS4 (Kopping et al., 2019). Overall, Thanassi et al. have demonstrated that the TolC, FtlC, and SilC systems have overlapping and distinct patterns of resistance, suggesting overlapping and distinct efflux functions (Kopping et al., 2019). New methods such as high-throughput screening systems to test for compounds that inhibit Francisella efflux pumps may advance this area of research (Haynes et al., 2018). Thus, these systems appear to represent the full complement of multidrug efflux pump systems in the genus *Francisella*.

Additional efflux systems have been identified which are not classified as multidrug efflux systems and are summarized below. Studying the annotated genes in *Francisella* in various databases suggests that the following genes may also be part of efflux pump systems for various metabolites including arsenite. A new antibiotic has been identified from soil bacteria, the organiarsenical arsinothricin, which targets glutamine synthetase. This antibiotic has been shown to be effective against Gram-negative and Gram-positive bacteria, although *Francisella* was not tested (Nadar et al., 2019). Interestingly, resistance to this antibiotic is mediated by *arsN1*, a gene in the bacterial arsenic resistance (ars) operons. While *arsN1* was not identified in *Francisella*, multiple genes have been found in this organism that are related to arsenite resistance, especially *arsRB*, where *arsB* is the arsenite-antimonite efflux family protein gene, and *arsR* is the arsenate/arsenite/antimonite-responsive transcriptional repressor gene. This pump system is responsible for the resistance to trivalent arsenite by its extrusion from bacterial cells and it contains the conserved 10 transmembrane domain structure with a conserved catalytic cysteine (Fu et al., 2009). The genes encoding this system have been well described in *F. philomiragia*, a related environmental strain (Siddaramappa et al., 2012). As one example, FTN_0382 is annotated as encoding a potential ArsB protein, while FTN_0395 is annotated as encoding a potential ArsR. Multiple winged helix-turn-helix transcriptional regulator proteins are identified as being related to ArsR in a search of NCBI/NLM database in *F. novidida* including FTN_0395, FTN_0801, FTN_0858, FTN_1022, FTN_1534, and FTN_1393. Two potential *arsB* genes are identified by this search, including FTN_0382 and FTN_0800. For example, an arsenic resistance locus has been putatively identified in *F. novicida* U112 by a computational approach, but not in all strains of *Francisella.* This locus includes *emrE* (FTN_0799), a gene which is annotated to be encoding a multidrug resistance antiporter of cations and cationic drugs; and *arsRB* (FTN_0800 and FTN_0801), two arsenite resistance genes, with *arsB* potentially being the arsenite efflux transporter and *arsR* potentially acting as the ArsR repressor (Siddaramappa et al., 2011). Of these, *emrE* (FTN_0799) and *arsB* (FTN_0800) were found to be required for *F. novicida* intracellular replication in U937 macrophages, while *arsR* (FTN_1393) was required for replication in *Drosophila* S2 cells (Asare and Abu Kwaik, 2010).

**Research Question:** In what ecosystem or lifecycle stage is *F. novicida* exposed to arsenite such that it potentially needs more than one arsenite resistance system? Does *Francisella* arsenite resistance cross-confer antibiotic resistance?

## Phenotypic Antibiotic Resistance in *Francisella* Due to Biofilm Formation

One consequence of the formation of biofilm in bacteria is the phenotypic resistance to antibiotics. This is thought to be due to the protective nature of the extracellular polymeric substance of the biofilm, rather than any direct genetically encoded resistance mechanism. It has recently been found that many species within the genus *Francisella* can form biofilms to lesser (Type A/B) or greater (environmental strains) extents (Dean et al., 2009, 2015, 2020; Durham-Colleran et al., 2010; Margolis et al., 2010; Verhoeven et al., 2010; Zogaj et al., 2012; van Hoek, 2013; Soto et al., 2015; Champion et al., 2019). The nature of the biofilm matrix in *Francisella* remains under study, but its formation appears to be induced by stress (Dean et al., 2009) and is regulated at least in some *Francisella* species by ppGpp/relA (Dean et al., 2009, 2015; Zogaj et al., 2012) and a two-component system *qseC/qseB* (Durham-Colleran et al., 2010). Inducing dispersal of the biofilm via chitinase or BDSF increases the susceptibility of *F. novicida* to antibiotics and antimicrobial peptides (Chung et al., 2014; Dean et al., 2015, 2020). It was also shown that biofilm mode of growth reduced fluoroquinolone susceptibility of *F. novicida*, further supporting the hypothesis of the mechanism of biofilm-mediated phenotypic resistance (Siebert et al., 2019).

A recently study of spontaneous ciprofloxacin- and streptomycin-resistant strains of *Francisella* had the interesting finding that all the *F. novicida* ciprofloxacin-resistant mutants had significantly less biofilm formation than the *F. novicida* wild-type parent strain, while the *F. novicida* streptomycin-resistant strains did not show this effect (Biot et al., 2020). Molecular analysis of the off-target mutations in the ciprofloxacin resistant strains did not reveal any obvious regulators of biofilm production or degradation, thus this biofilm effect may be due to a general increase in stress for these mutants, indicated by their reduced fitness (Biot et al., 2020).

## New Methods to Assess Antimicrobial Resistance of *Francisella* as an Intracellular Pathogen

Determining which antibiotic is most effective for treatment of a patient has always been challenging. Mimicking all *in vivo* conditions is impossible and persistent use of model animals impractical. Thus, methods such as MIC, minimum biocidal concentration (MBC), minimal concentration effective against biofilm (MCEB), and Kirby Bauer (specialized disk diffusion assay) have all been used to address these questions *in vitro*. While these methods are relatively inexpensive as well as amenable to high throughput, they lack *in vivo* characteristics to better confirm the efficacy of any antimicrobial against a particular pathogen.

### Activity in the Presence of Serum

*Francisella* spends some portion of its lifecycle in a host, outside of a phagocytic cell, where it is subject to opsonization and uptake for destruction by immune cells. Thus, there is an opportunity for antimicrobial agents to encounter the bacterium in the blood or in the tissue, external to host cells. One way to better model *in vivo* response of the bacteria to any antibacterial agent may be to perform the MIC assays in the presence of serum, or even a serum bactericidal titer (SBT) assay (Zaghi et al., 2020). The presence of serum can model some aspects of antibiotic pharmacodynamics, and can directly affect the activity of antibacterial compounds such as peptides. An assay was developed using plasma from patient samples along with immunomagnetic separation to enhance detection (Aloni-Grinstein et al., 2017). Such approaches to improve detection of activity could improve the development of new potential therapeutics.

### Intracellular MIC Assay

These problems of translating *in vitro* MIC results to *in vivo* clinical outcomes are magnified when regarding intracellular bacteria as the penetration, and local concentrations of antimicrobials within eukaryotic cells become extremely important. Researchers have developed intracellular MIC assays in which bacterial cells are phagocytosed by eukaryotic host cells, extracellular bacterial cells are killed by exogenous addition of antibiotic such as gentamicin, and then different populations of infected eukaryotic cells received different doses of a drug, similar to an MIC. After a time for incubation (18–24 h), bacterial cells are quantified either via eukaryotic cell lysis and cell plating (if possible) or a variety of other quantification methods based upon the bacterium in question. Such an assay was performed to demonstrate the synergistic activity of gallium to gentamicin against intracellular *F. tularensis* SchuS4 (Lindgren and Sjostedt, 2016). This method has been performed with *Francisella* and shows reasonable predictive power of patient outcome when compared to clinical data (Maurin et al., 2000). A more recent method developed by Sutera et al. involves a dye uptake assay to quickly and quantitatively measure the susceptibility of intracellular *Francisella* to extracellular antibiotics. If the bacteria are inhibited by the extracellular antibiotic, then they will have reduced intracellular replication and reduced host cell lysis compared to untreated controls. This method of staining for eukaryotic cell viability also included a focus on *Francisella* virulence factors as the bacterial cells need to not only enter the phagosome, escape to cytoplasm, and survive but lyze the eukaryotic cells for the eukaryotic cell measurement (via neutral red) to be reduced (Sutera et al., 2014a).

### Insect Models

Insect models have recently been used to test the efficacy of antibiotics against Francisella. These *in vivo* models allow for the more complex conditions of a host to be incorporated into the assay. While ticks, biting flies, and mosquitoes are known insect vectors for this disease, recently published insect models for *Francisella* virulence include *Drosophila melanogaster* (Ahlund et al., 2010; Moule et al., 2010), the orange spotted cockroach (Eklund et al., 2017), and the wax worm larvae, *Galleria mellonella* (Aperis et al., 2007; Sprynski et al., 2014; Propst et al., 2016b; Thelaus et al., 2018; Djainal et al., 2020). The correlation between virulence factors in humans and virulence in the waxworm is not exact (Thelaus et al., 2018), thus this model needs to be used with care in interpretation of the results, especially with respect to host-directed virulence. However, if an infection is successfully established, the waxworm is a quick method of testing antibacterial activity of test compounds. The waxworm has been used as an insect model for antibiotic treatment studies for *Francisella*, demonstrating the activity of azithromycin and the prodrug of fosmidomycin, for example (Ahmad et al., 2010; McKenney et al., 2012), as well as ciprofloxacin, levofloxacin, or streptomycin (Aperis et al., 2007).

### *Francisella* qPCR-Based Antibiogram

A recently developed method both addresses the intracellular antibiotic susceptibility problem and provides a much faster test method. Aloni-Grinstein et al. developed a method of *Francisella* antibiogram which uses qPCR and was able to confirm that gentamicin does not inhibit intracellular *Francisella* replication while other antibiotics such as doxycycline, chloramphenicol, and ciprofloxacin showed intracellular activity, consistent with results from both *in vitro* and clinical studies (Aloni-Grinstein et al., 2015). This rapid 3-h method was shown to provide a quantitative measure of minimal inhibitory extracellular concentration (MIEC), which is an important measure of the amount of extracellular antibiotic needed to eradicate intracellular *Francisella* bacteria. The authors were able to examine the results of the gentamicin studies and pose the question as to whether gentamicin is suitable for clinical use against tularemia given this lack of inhibition of intracellular replication (Aloni-Grinstein et al., 2015).

### Use of Transport Mutants to Identify Lipophilic Drug Candidates

**FIGURE 3:**
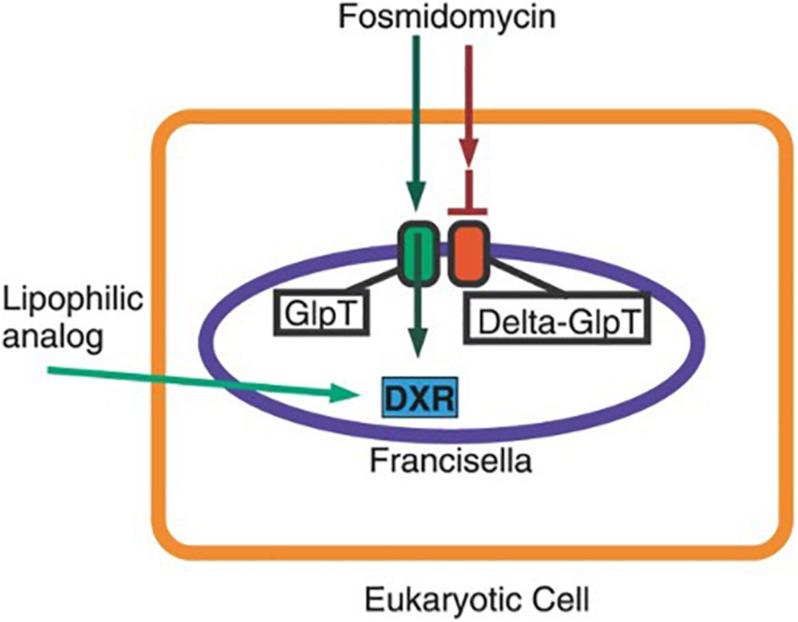
Model for screening method to identify lipophilic, fosmidomycin-derived analogs effective against intracellular pathogens. Figure used under Creative Commons Attribution (CC BY 4.0) license (McKenney et al., 2012). In this system, the mammalian cell (orange line) is infected with intracellular bacteria (purple line), *F. novicida* (green GlpT protein), or *F. novicida glpT* mutant (red GlpT protein) separately. We demonstrated that the drug fosmidomycin is dependent on GlpT transporter to reach its intracellular target (DXR) in *Francisella.* This model demonstrates how we can use a mutant in a drug transporter in *Francisella* (blue cell) to screen for fosmidomycin analogs and prodrugs that act (i) independently of that transporter and (ii) are lipophilic and (iii) able to pass through both a bacterial membrane and (iv) a eukaryotic host cell membrane. Shown here is the difference between the parent compound fosmidomycin, which can pass through the host cell membrane, but requires *Francisella* GlpT to enter the bacteria, vs a lipophilic analog, which can pass through the host cell membrane and the bacterial cell membrane and can act on the drug target (DXR). The library of fosmidomycin analog candidates was first generated by testing candidate molecules against recombinant purified *Francisella* DXR *in vitro* (Jawaid et al., 2009). This intracellular model is constructed by infecting host cells with a glpT mutant of *Francisella*, and then screening a library of fosmidomycin analogs for inhibition of *Francisella* growth comparing the wild-type *F. novicida* vs the *glpT* mutant *F. novicida* for intracellular replication (*123*).

Finally, through the use of mutant bacteria from the transposon mutant library in *F. novicida* (Gallagher et al., 2007), intracellular screening assays can be rapidly set up to directly address certain experimental constraints. For example, in our screening for a fosmidomycin-related compound, we sought a compound whose antibacterial activity against intracellular *Francisella* was not dependent on the glpT transporter, which transported the non-lipophilic fosmidomycin prodrug into the bacterial cells. Infecting cells with a glpT transposon mutant of *F. novicida*, we were able to screen for compounds that inhibit *Francisella* intracellular growth without dependence on the bacterial glpT transporter for entry into the bacterium (Jawaid et al., 2009; McKenney et al., 2012). This screen applied the criteria of the drug being able to cross both the eukaryotic and the prokaryotic membrane, building in a powerful selection step in the screening method. This provided a very rapid, direct, and powerful screen for lipophilic versions of compounds that can act on intracellular *Francisella* in a glpT-independent manner, and for a compound which has the property of crossing both the eukaryotic and prokaryotic membranes as shown in Figure 3.

**Research Question:** What other antibiotics can affect the replication of intracellular *Francisella*? The intracellular nature of the infection should be emphasized when exploring antibiotic resistance or sensitivity of *Francisella* to new compounds.

## Contribution of Antibiotic Resistance to Bacterial Fitness in *Francisella*

The possibility of either the natural or engineered emergence of AMR in the bacterial biothreat agents has always been of concern. Central to this concern is the fitness of organisms carrying such AMR characteristics and their ability to infect and replicate in the host. Recently, a study was undertaken by Biot et al. (2020) to assess the fitness of spontaneous ciprofloxacin- and streptomycin-resistant *F. novicida* and *F. holarctica* LVS. Fitness was assessed through intracellular replication in host macrophages as well as through *in vivo* infection in mice. This study found that the ciprofloxacin-resistant strains in both parental backgrounds were attenuated in J774A.1 murine macrophage cells and caused no infection in BALB/c mice at any concentration of bacteria tested. This may reflect the stress that the DNA Gyrase mutations place upon the organism, which may be detrimental to its overall survival (and also affected biofilm production). In contrast, they demonstrated that the streptomycin-resistant strains in the LVS background were not significantly attenuated in macrophage cells (although more attenuated in the *F. novicida* background) and were attenuated in BALB/c mice but still able to cause death, perhaps reflecting the lesser centrality of the streptomycin drug target in *Francisella*.

## Conclusion

As new antibiotic development is exceedingly slow and *Francisella* is only susceptible to a few clinically relevant antibiotics, the continual monitoring of *Francisella* for enhanced or emerging AMR is essential. We have highlighted the many ways that *Francisella* resists current antimicrobials, as well as methods *Francisella* may employ to become more resistant to them. We also reviewed the new approaches to assessing the antimicrobial susceptibility of *Francisella* when it is intracellular, which may provide a relevant model for the *in vivo* situation. Areas of encouragement for further study were highlighted in Research Questions.

Drug development against all Gram-negative bacteria is an urgent need in light of the emerging AMR crisis, and work done of some of the more intractable and intracellular Gram-negative organisms should help identify novel antimicrobial agents which may prove effective against a broad range of pathogens.

## Methods

### Literature Search

Google Scholar was used to search for original reports of genetic mechanisms of AMR in *Francisella* (1950 to November 2020). We focused on *tularensis*, *holarctica*, *novicida*, and *hispaniensis* sub-strains. Search phrases used included “antibiotic resistance,” “multidrug resistant,” “beta-lactam resistance,” “polymyxin resistance,” “erythromycin resistance,” “macrolide resistance,” “quinolone resistance,” “fosmidomycin resistance,” and “antibiotic resistance intracellular determination.” All were appended to “*Francisella*.” In addition, literature searches through PubMed^1^ were done with the terms “*Francisella*” AND the relevant antibiotic AND “resistance.” All genomic, proteomic, *in vitro*, *in vivo*, and intracellular growth data were considered.

### Database Search

Uniprot^2^ was used to identify groups of relevant genes or proteins in various *Francisella* strains. Pubmed Gene^3^ and Protein database^4^ searches were done with the terms “*Francisella*” AND the relevant antibiotic AND “resistance,” or “*Francisella*” and “efflux pumps,” or “*Francisella*” and “penicillin-binding proteins,” etc. The KEGG genes database^5^ and the PGAT tool (Brittnacher et al., 2011) were also searched for similar terms. Each of these strategies was employed for each type of antibiotic resistance.

## Author Contributions

MvH conceived the study. SK and MvH wrote and edited the manuscript. Both authors approved the final version of the manuscript.

## Conflict of Interest

The authors declare that the research was conducted in the absence of any commercial or financial relationships that could be construed as a potential conflict of interest.

## Footnotes

1 ncbi.nlm.nih.gov

2 http://www.uniprot.org

3 https://www.ncbi.nlm.nih.gov/gene/

4 https://www.ncbi.nlm.nih.gov/protein/

5 https://www.kegg.jp/kegg/genes.html
